# Antioxidant and Antimicrobial Biofoil Based on Chitosan and Japanese Knotweed (*Fallopia japonica*, Houtt.) Rhizome Bark Extract

**DOI:** 10.3390/antiox11061200

**Published:** 2022-06-18

**Authors:** Katerina Naumoska, Urška Jug, Kristi Kõrge, Ana Oberlintner, Majda Golob, Uroš Novak, Irena Vovk, Blaž Likozar

**Affiliations:** 1 Laboratory for Food Chemistry, Department of Analytical Chemistry, National Institute of Chemistry, Hajdrihova 19, 1001 Ljubljana, Slovenia; irena.vovk@ki.si; 2 Department of Catalysis and Chemical Reaction Engineering, National Institute of Chemistry, Hajdrihova 19, 1001 Ljubljana, Slovenia; kristi.korge@ki.si (K.K.); ana.oberlintner@ki.si (A.O.); uros.novak@ki.si (U.N.); blaz.likozar@ki.si (B.L.); 3 Institute of Microbiology and Parasitology, Veterinary Faculty, University of Ljubljana, Gerbičeva ulica 60, 1000 Ljubljana, Slovenia; majda.golob@vf.uni-lj.si

**Keywords:** Japanese knotweed rhizome bark extract, chitosan, antioxidant biofoil, antimicrobial activity, biodegradable material, active packaging

## Abstract

A 70% ethanol_(aq)_ extract of the rhizome bark of the invasive alien plant species Japanese knotweed (JKRB) with potent (in the range of vitamin C) and stable antioxidant activity was incorporated in 1% *w/v* into a chitosan biofoil, which was then characterized on a lab-scale. The 2,2-diphenyl-1-picrylhydrazyl (DPPH) assay confirmed the antioxidant activity of the JKRB biofoil upon contact with the food simulants A, B, C, and D1 (measured half-maximal inhibitory concentrations—IC_50_) and supported the Folin–Ciocalteu assay result. The migration of the antioxidant marker, (−)-epicatechin, into all food simulants (A, B, C, D1, D2, and E) was quantified using liquid chromatography hyphenated to mass spectrometry (LC-MS). Calculations showed that 1 cm^2^ of JKRB biofoil provided antioxidant activity to ~0.5 L of liquid food upon 1 h of contact. The JKRB biofoil demonstrated antimicrobial activity against Gram-positive bacteria. The incorporation of JKRB into the chitosan biofoil resulted in improved tensile strength from 0.75 MPa to 1.81 MPa, while elongation decreased to 28%. JKRB biofoil’s lower moisture content compared to chitosan biofoil was attributed to the formation of hydrogen bonds between chitosan biofoil and JKRB compounds, further confirmed with attenuated total reflectance Fourier transform infrared spectroscopy (ATR-FTIR). The JKRB biofoil completely degraded in compost in 11 days. The future upscaled production of JKRB biofoil from biowastes for active packaging may support the fights against plastic waste, food waste, and the invasiveness of Japanese knotweed, while greatly contributing to the so-called ‘zero-waste’ strategy and the reduction in greenhouse gas emissions.

## 1. Introduction

Japanese knotweed (*Fallopia japonica* Houtt. or *Polygonum cuspidatum* Siebold & Zucc.) is an invasive alien plant species which was brought from East Asia to Europe in the 19th century as an ornamental plant. Due to its rapid spread and its resistance to extermination, today, it causes great ecological and economic damage in Europe and North America [1].

The plant’s underground biomass corresponds to up to 2/3 of the total biomass. Its characteristic high growth rate (up to 15 cm/day) enables rapid domination in the areas in which it grows. As Japanese knotweed’s reproduction is mostly vegetative, it only takes a small fragment of a rhizome or a stem for a new individual to grow. Due to climate change (increased temperatures and decreased numbers of early frost days), it is predicted to spread to higher altitudes and latitudes [1].

The plant alters soil composition, displaces indigenous plant species, and affects the physical and chemical quality of watercourses, causing major damage to infrastructure [1]. Accordingly, the annual economic damage caused by Japanese knotweed in Great Britain is estimated to be EUR 230 million, out of EUR 2.3 billion in the whole of Europe [1]. Its spread could be limited through several eradication techniques, i.e., mechanical (excavation or incineration), chemical (with herbicides), and biological (with mites, nematodes, and fungi); however, these have been shown to be economically and/or environmentally unsustainable techniques [1]. Therefore, the exploitation of Japanese knotweed rhizomes, which will allow for the utilization of biowaste for beneficial purposes, would favor mechanical over other eradication techniques.

In contrast to its negative impact on the environment, the rhizomes of Japanese knotweed are widely used in traditional Chinese medicine due to their positive effects on human health. The extracts and isolated compounds of Japanese knotweed have shown antioxidant, antibacterial, anticancer, antiproliferative, apoptotic, anti-inflammatory, and antiviral effects [2]. The rhizomes represent a source of a variety of pharmacologically active metabolites, especially stilbenes, anthraquinones, flavan-3-ols, and B-type proanthocyanidins, phenolic acids, triterpenic acids, as well as naphthalenes and compounds of other phytochemical groups [3,4,5,6,7]. The rhizome bark of Japanese knotweed is considered to be the richest part of the rhizome phytochemically. The rhizomes and roots of Japanese knotweed are included in the 9th and 10th editions of the European Pharmacopoeia in the chapter “Herbal drugs and herbal drug preparations” [8,9].

The antioxidant activity of the Japanese knotweed rhizome has been confirmed using various tests [4,10,11,12,13,14,15,16]. The correlation between the antioxidant activity of the extract and the proanthocyanidins has been observed by using principal component analysis [7]. On the other hand, dietary supplements of Japanese knotweed rhizomes that promote *trans*-resveratrol as its main antioxidant are present on the market.

In a recent study [17], a DPPH assay was used to prove that various Japanese knotweed rhizome bark extracts possess strong antioxidant activity, equivalent to that of ascorbic acid (vitamin C). The advantage of the 70% ethanolic_(aq)_ extract compared to ascorbic acid is the stability of its antioxidant activity over time. The antioxidant activities of the Japanese knotweed rhizome bark extract (IC_50_~3.70 µg mL^−1^) and the compound proven to be responsible for its antioxidant activity, (−)-epicatechin (IC_50_~1.60 µg mL^−1^), were stable for at least 14 days, while the antioxidant activity of ascorbic acid decreased significantly over time (IC_50_: 3.12–62.79 µg mL^−1^) [17].

The antibacterial activity of Japanese knotweed rhizome extracts has already been tested on five common foodborne pathogens (*Escherichia coli*, *Salmonella enterica* subsp. *enterica* serovar Anatum, *Listeria monocytogenes*, *Bacillus cereus*, and *Staphylococcus aureus*). The extracts have thus shown great potential for use as natural food preservatives [2,18].

Addressing invasive alien species was one of the key objectives of the EU’s 2020 Biodiversity Strategy [19]. Moreover, the European Commission’s top priority is to replace the use of single-use plastics with biodegradable products, reduce carbon footprints, and increase the re-use of products in the circular economy [20].

Active packaging can prolong the shelf life of fast-spoiling food compared to conventional packaging [21], consequently reducing the amount of food waste. The solution might be hidden in 6–8 million tons of biowaste rich in chitin, the second most common polysaccharide on Earth [22], produced annually in the food-processing industry involving crustaceans [23]. Biodegradable foils intended for use in food, cosmetics, and pharmaceutical packaging can be prepared on the basis of chitosan, a derivative of chitin, which has huge potential due to its ubiquitous and inexpensive source [22], compostability [24], wide field of applications, non-toxicity, biocompatibility, good ability to form foils and antimicrobial activity [23]. To be comparable and even go beyond conventional petrol-based packaging, the improvement of mechanical, antimicrobial, and antioxidant properties, as well as water vapor and gas barrier properties, is achieved with the incorporation of bioactive plant extracts or compounds [25,26,27,28,29].

Numerous natural extracts with antioxidant (e.g., extracts of green tea, mint leaves, pomegranate peel, grape seed, grapefruit seed, rosemary, sage, oregano, basil, ginger, propolis, cinnamon, thyme, ginseng, ginkgo leaf, ginger, barley husk, oak, hop, and chestnut) [21,23,30,31,32,33,34] and antimicrobial properties (e.g., extracts of grape seed, mint, pomegranate peel, betel leaf, green tea, propolis, turmeric, apple peel, citrus, oak, clove, coriander, marjoram, cinnamon, and cumin) [23,30,32,34] have been used for the formulation of active packaging. A huge number of these extracts (e.g., extracts of green tea, grape seed, rosemary, propolis, turmeric, apple peel, citrus, hop, chestnut, and pomegranate peel) were incorporated into a chitosan biopolymer [23,30,31,32,34].

To date, no study has elaborated the incorporation of Japanese knotweed extract into a (bio)polymer material for the formulation of a biodegradable foil for food packaging or any other applications.

This study presents a pioneer research work on the active packaging prepared using the underutilized, under-investigated, and highly available natural resources of the invasive plant species Japanese knotweed, rich in various antioxidants.

The aims of the present study were as follows: (1) to develop chitosan-based biodegradable foil, enriched with a highly potent antioxidant extract of Japanese knotweed rhizome bark; (2) to evaluate biofoil’s properties (physicochemical, antimicrobial, and antioxidant properties and biodegradability) relevant for the packaging industry; (3) to study the migration of the antioxidant marker (−)-epicatechin into food simulants which will enable classification of the biofoil into non-migratory or migratory active packaging. The future upscaled production of such materials would make a significant contribution to a waste-free society, reducing environmental pollution caused by conventional plastics and reducing greenhouse gas emissions through the use of biowaste. Moreover, the economic and ecological issues associated with the growth of the invasive Japanese knotweed plant would be solved by its excavation. The combination of the two biowastes: 1. chitosan—a natural biopolymer derived from crustacean waste (e.g., shrimps) and 2. Japanese knotweed rhizome bark—a leftover plant material obtained after the plant’s excavation will allow the formulation of low-cost biofoils with high added value.

## 2. Materials and Methods

**Figure 1:**
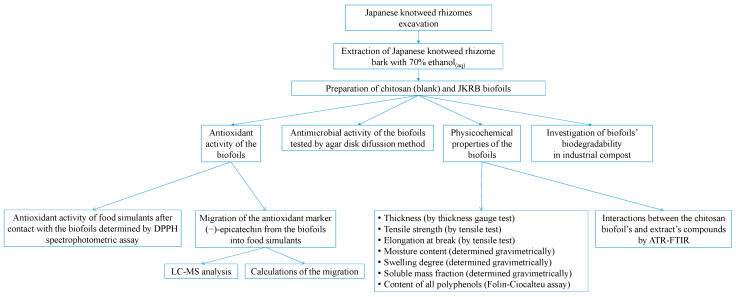
A flow chart of work performed throughout the study.

Figure 1 depicts a flow chart of work performed throughout the study.

### 2.1. Chemicals

All solvents used during the experimental work were at least of analytical grade. Methanol (HPLC and LC-MS grade) and acetonitrile (HPLC and LC-MS grade) were purchased from Honeywell Reagents (Seelze, Germany). Ethanol (anhydrous, absolute) was purchased from Carlo Erba Reagents (Val de Reuil, France). Acetic acid (glacial 100%) and acetic acid (100%) for LC-MS were obtained from Merck (Darmstadt, Germany). Standard (−)-epicatechin (90%), 2,2-diphenyl-1-picrylhydrazyl (DPPH), Tenax^®^ porous polymer adsorbent (60−80 mesh, pore size 200 nm), gallic acid, and Folin–Ciocalteu’s phenol reagent were supplied by Sigma-Aldrich (Steinheim, Germany). Ultrapure water was provided by the Milli-Q ultrapure water preparation system (18 MΩ cm^−1^; Millipore, Bedford, MA, USA). LC-MS-grade solvents were used for the preparation of the LC-MS mobile phases, (−)-epicatechin and JKRB solutions for LC-MS, as well as for the extraction of Tenax^®^. Disposable plastic cuvettes were purchased from Brand (Wertheim, Germany).

High-molecular-weight chitosan (deacetylation rate < 75%, 310–375 kDa) and lactic acid were purchased from Sigma-Aldrich (Steinheim, Germany), and glycerol was obtained from Pharmachem Sušnik (Ljubljana, Slovenia).

### 2.2. Extraction of Japanese Knotweed Rhizome Bark

Japanese knotweed (*Fallopia japonica* Houtt.) rhizomes were harvested in Ljubljana, Slovenia (Vrhovci, by a bridge over Mali Graben, N 46°02′33.9″; E 14°27′00.9″). A voucher specimen was deposited in the Herbarium LJU (LJU10143477). The rhizomes were washed with tap water and dried on a paper towel at room temperature. Peeled rhizome bark was lyophilized at –50 °C for 24 h. The dry material was then frozen with liquid nitrogen and pulverized with a Mikro Dismembrator S with a frequency of 1700 min^−1^ (1 min). Lyophilized and pulverized Japanese knotweed rhizome bark material (2 g) was weighed and extracted twice with 70% ethanol_(aq)_ (1st 40 mL; 2nd 20 mL), vortexed (5 min), sonicated in an ultrasonic bath (15 min), and centrifuged (6700× *g*, 5 min). The supernatant of both extractions was transferred to pre-weighed storage vials, the solvent was removed with a stream of nitrogen, and the vials containing dry extract (JKRB) were weighed to calculate the mass of the obtained dry extract and the yield of extraction.

### 2.3. Preparation of Chitosan-Based Biofoil with and without Incorporated Japanese Knotweed Rhizome Bark Extract

The preparation of the film-forming solutions (FFSs) for the biofoil formulation and the testing of their physicochemical properties was conducted in accordance with the protocol described in the literature [35]. Chitosan (1.5%, *w/v*) and glycerol (plasticizer; in 30% according to the mass of chitosan) were completely dissolved in aqueous lactic acid (1.0%, *v/v*) and stirred on a magnetic stirrer (IKA, Staufen, Germany) at 1000 min^−1^ at room temperature (24 °C) until the homogeneity of the suspension was achieved. The suspension was cleaned from all major impurities by filtering through medical gauze. The extract of Japanese knotweed rhizome bark (1% JKRB, *m/v*) was added to half of the clear solution. The other part of the solution was used for the formulation of chitosan biofoil, which served as a blank sample in the analyses. The FFSs with the added JKRB were homogenized (2 min, at 6000 min^−1^) with Ultra-Turrax T50 (IKA, Staufen, Germany), and the solutions were left overnight. The next day, they resulted in some sticky foam, which was carefully removed with a spatula.

The FFSs were poured into rectangular 12 cm × 12 cm Petri dishes made of polyurethane (~0.32 mL FFS per cm^2^ Petri dish) and allowed to dry at a laminar flow at an air flow rate of 60 m^3^ h^−1^ (Microbium d.o.o., Ljubljana, Slovenia) for 24 h. The obtained chitosan-based biofoils with and without incorporated JKRB (further referred to as chitosan biofoil and JKRB biofoil) were scraped from Petri dishes, separated from each other with baking paper to prevent direct contact of the biofoil pieces, and stored in an airtight container (24 °C, protected from light) before the analyses.

### 2.4. Antioxidant Activity of the Biofoils

#### 2.4.1. DPPH Scavenging Activity of Food Simulants after Contact with the Biofoils

The chitosan (blank) and JKRB biofoils were cut in circular pieces with diameters of 5.5 mm and separately exposed (about 77 mg) to 20 mL of different liquid food simulants (10% ethanol_(aq)_ (*v/v*), 3% acetic acid (*w/v*), 20% ethanol_(aq)_ (*v/v*), 50% ethanol_(aq)_ (*v/v*), and 95% ethanol_(aq)_ (*v/v*), also known as food simulants A, B, C, D1, and D2, respectively), selected according to the European Commission’s document [36].

**Figure 2:**
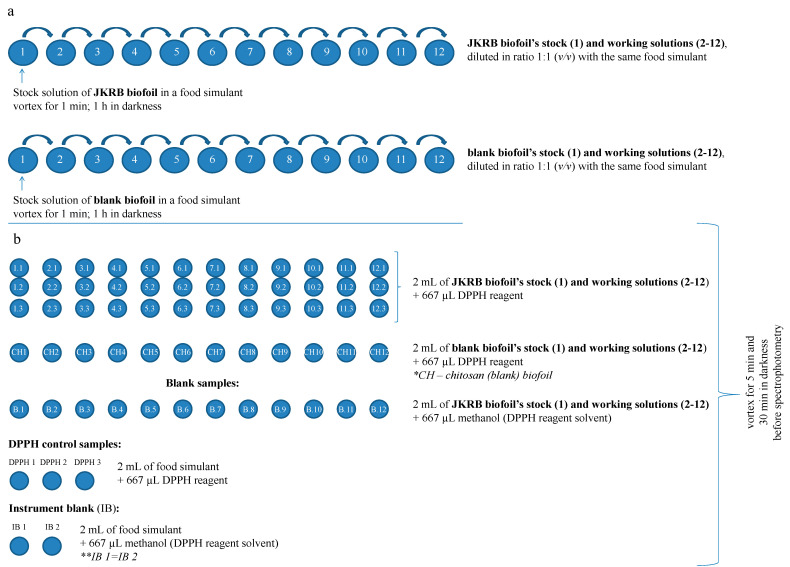
Scheme of the DPPH antioxidant assay: (**a**) preparation of stock and working solutions, (**b**) reactions before spectrophotometric measurements.

The liquid food simulants containing the pieces of JKRB and blank biofoils were vortexed for 1 min and left in the dark in closed glass containers for 1 h to allow the biofoil compounds to leach into the contact liquid. Biofoil pieces were then removed, and the contact liquid (stock solution: approximately 3.85 mg mL^−1^ mass of the biofoil relative to the volume of contact liquid) was diluted with the same food simulant in a ratio of 1:1 *v/v* to obtain nine (in the case of 20% and 50% ethanol_(aq)_ (*v/v*)) or eleven working solutions (in the case of other liquid simulants) (Scheme a in Figure 2).

Each stock and working solution (2 mL) was transferred in triplicate (in the case of JKRB biofoil) or in one parallel (in the case of blank biofoil) into another vials to which DPPH reagent (667 µL of 200 µM methanolic solution of DPPH) was immediately added (solutions labeled with _A_ in Equation (1) [37]; Scheme b in Figure 2). To subtract the absorbance corresponding to the color of the JKRB biofoil leachates from the absorbance corresponding to the positive DPPH reaction color, blank samples were also prepared in one parallel, so that to 2 mL of stock and working solutions, 667 µL of methanol (reagent solvent) was added (solutions labeled with _B_, Equation (1) [37]; Scheme b in Figure 2). For DPPH control samples, 2 mL of food simulant was mixed with 667 µL of DPPH reagent (solutions labeled with _C_, Equation (1) [37]; Scheme b in Figure 2). An instrument blank (2 mL of food simulant mixed with 667 µL of methanol) was also prepared (Scheme b in Figure 2).

**Figure 3:**
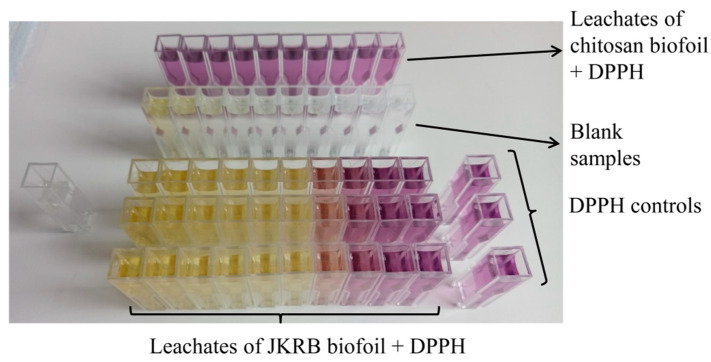
An example of a DPPH assay executed on chitosan (blank) and JKRB biofoils using 50% ethanol_(aq)_ as a food simulant. Different dilutions of leachates of JKRB biofoil to which DPPH was added were tested in three parallels. Different dilutions of leachates of chitosan (blank) biofoil with exposure to DPPH reagent were tested in one parallel. Blank samples (leachates of JKRB biofoil to which reagent solvent—methanol was added) were also measured to subtract the absorbance corresponding to the color of the JKRB biofoil leachates themselves. Moreover, a blank for the spectrophotometer and 3 DPPH controls were prepared.

All mixtures were prepared in amber glass vials, vortexed for 5 s, and kept in darkness at room temperature for 30 min. The absorbances (A_A_, A_B,_ and A_C_) of solutions _A_, _B_, and _C_ placed in disposable plastic cuvettes were measured spectrophotometrically at 517 nm (Lambda 45 UV/Vis spectrophotometer) (Figure 3). GraphPad Prism 9 was used for IC_50_ calculations and curve plotting [38].

DPPH scavenging effect (%) = 100 − ((A_A_ − A_B_) × 100/A_C_) (1)

A_A_ = absorbance “stock/working solution + DPPH reagent”;

A_B_ = absorbance “stock/working solution + methanol (reagent solvent)”;

A_C_ = absorbance “food simulant + DPPH reagent”.

#### 2.4.2. Migration of the Antioxidant Marker, (−)-Epicatechin, from the Biofoils into Food Simulants

##### LC-MS Analysis

Food simulants separately exposed to chitosan (blank) and JKRB biofoils were analyzed with the UHPLC-MS system (Dionex Ultimate 3000−LCQ Fleet, Thermo Scientific, San Jose, CA, USA), using a previously developed reversed phase (RP)-HPLC-MS method [17]. Heated electrospray ionization (HESI) in the negative ion mode was used for the ionization of the compounds. Some minor modifications, as described below, were applied, as for this study, another LC-MS system was used. A column cleaning and re-equilibration phase was introduced before each injection as stated: 100% B (30–40 min); 100–10% B (40–41 min); 10% B (41–45 min). The MS parameters were the same as in the reported method, except the capillary voltage was −45 V, and the tube lens was −125. The MS spectra were recorded in the *m/z* range of 50–2000. The mass peak of (−)-epicatechin at *m/z* 289 was fragmented in MS^2^ using a collision energy of 30%, while spectra in the SIM mode were acquired at *m/z* 289.00.

Xcalibur software (version 4.0.27.42, Thermo Fisher Scientific, Breda, The Netherlands) was used to evaluate the collected chromatograms and spectra. The presence of (−)-epicatechin in food simulants was confirmed using the (−)-epicatechin standard. The method was employed to identify and quantify (−)-epicatechin leached in food simulants after 1 h of contact with the biofoils as well as in JKRB.

Except the stock solution prepared with food simulant D2, which was 3-fold concentrated before the analysis, all other stock solutions prepared with the liquid food simulants (Section 2.4.1.) were directly injected into the LC-MS. Tenax was also tested as a dry food simulant (also known as simulant E) [36]. Chitosan and JKRB biofoils were cut into 2 cm × 2 cm pieces in triplicate. Each piece was covered with 0.16 g of Tenax powder (4 g of Tenax dm^−2^, [36]), wrapped with Alu foil and placed on Petri dishes in an oven at 60 °C for 10 days to simulate the ‘’long-term’’ (longer than 6 months) storage of dry food. After the aging experiment, Tenax powder which was in contact with the biofoils was extracted with 3 mL of methanol, vortexed for 5 min, ultrasonicated for 15 min, and centrifuged at 6700× *g* for 5 min. The proportion of Tenax: methanol was taken from the literature [39]. The extraction was repeated twice, and the supernatants were combined, filtered through 0.2 µm hydrophilic polytetrafluoroethylene (H-PTFE) filters (Macherey-Nagel, Düren, Germany), and dried under N_2_ flow. The dry residues were kept in the freezer at −20 °C and dissolved in 1 mL of methanol before LC-MS analyses. For quantification purposes, the solutions of the three Tenax replications were pooled together, and the extract was then 10-fold concentrated.

For quantitative LC-MS analysis, the dry JKRB, prepared as described in Section 2.2., was dissolved in methanol to obtain a stock solution of 13.51 mg mL^−1^, and by its further dilution, a working solution of 1.35 mg mL^−1^.

For the construction of the calibration curve, (−)-epicatechin standard dissolved in methanol was prepared in the following concentrations (µg mL^−1^): 25.00, 20.00, 15.00, 10.00, 5.00, 2.50, 2.00, 1.00, 0.75, 0.50, and 0.25. The limit of detection (LOD), limit of quantification (LOQ), % relative standard deviation (RSD) of the areas of (−)-epicatechin standard peak at 2.5 µg mL^−1^ (*n* = 6), R^2^, range, accuracy, and precision were determined. The LOD and LOQ were calculated as concentrations of (−)-epicatechin (*n* = 6), resulting in a signal-to-noise ratio of at least 3 and 10, respectively. Due to the relatively simple matrix of the leachates, the stock solution obtained after 1 h of contact between chitosan (blank) biofoil and 3% acetic acid was dried under nitrogen and spiked with (−)-epicatechin standard dissolved in methanol at three levels (0.50 µg mL^−1^, 2.50 µg mL^−1^, and 5.00 µg mL^−1^). Accuracy was calculated as the % of the recovered amount measured for spiked samples against standard solutions (*n* = 3) of respective concentrations. Precision was also determined at all three levels using the same spiked samples and expressed as % RSD (areas; *n* = 6).

##### Calculations of the Migration of the Antioxidant Marker, (−)-Epicatechin

To calculate the % of migration of the antioxidant marker, (−)-epicatechin, from the biofoil into the food simulants after 1 h of contact, the area of one circle (5.5 mm diameter) of 0.24 cm^2^ was taken into account. It was also considered that for each stock solution containing JKRB biofoil (prepared for the determination of the antioxidant activity), about 20 circles with an approximate mass of 77 mg were added into 20 mL of a suitable solvent. From here, the calculations were as follows: (1) 46.11 mL of FFS was contained in 144 cm^2^ (FFS was poured into a 12 cm × 12 cm Petri dish with 0.32 mL of FFS cm^−2^); (2) 46.11 mL of FFS (in the 12 cm × 12 cm Petri dish) contained 0.46 g of JKRB (1% *w/v* JKRB was used for the formulation of the biofoil); (3) calculation through the area of the biofoil used to prepare stock solutions: if 0.24 cm^2^ was the area of one circle, 20 circles (used to prepare stock solutions) had an area of 4.74 cm^2^; as 144 cm^2^ contained 0.46 g of JKRB, 4.74 cm^2^ of biofoil contained 15.17 mg of JKRB. Therefore, the value of 15.17 mg of JKRB, present in the JKRB biofoil added to each stock solution, was taken for further calculations. (−)-Epicatechin % in the JKRB extract was determined using the LC-MS method. From here, the mass of (−)-epicatechin in the JKRB biofoil present in each stock solution was calculated. The concentration of (−)-epicatechin (µg mL^−1^) leached into some food simulants upon 1 h of contact with JKRB biofoil was also determined with the LC-MS method using the stock solutions and was further averaged for the antioxidant food simulants (measured IC_50_) and recalculated for 20 mL of solvent (volume of solvent present in the stock solution). Leached (−)-epicatechin (% leached out of 100% incorporated (−)-epicatechin) was therefore calculated.

To predict the approximate amount of liquid food to which 1 cm^2^ of biofoil provides antioxidant activity (in the range of measured IC_50_), the following data were taken into account: 4.74 cm^2^ of biofoil (calculated through the area of the biofoil) was equal to 20 mL of solvent used to prepare the stock solutions. From here, if 4.74 cm^2^ of biofoil was placed into 20 mL of solvent to prepare the stock solutions, 1 cm^2^ of biofoil would be placed into 4.2 mL of solvent. Further calculations were performed after obtaining the experimental results.

### 2.5. Antimicrobial Activity of the Biofoils

The antimicrobial activities of the chitosan (blank) and JKRB biofoils were assessed with the agar disk diffusion test method using different reference Gram-negative and Gram-positive strains from the internal bacterial collection at the Institute of Microbiology and Parasitology, Veterinary Faculty, University of Ljubljana. The potential antimicrobial activity of the biofoils was investigated against foodborne bacterial strains: *Escherichia coli* (RDK 052, WDCM 000013), extended-spectrum beta-lactamase (ESBL) producing *E. coli*, *Yersinia enterocolitica* (RDK 058, WDCM 00038), *Salmonella enterica* subsp. *enterica* serovar Typhimurium (RDK 054, WDCM 000031), *Salmonella enterica* subsp. *enterica* serovar Enteritidis (RDK 055, CAPM 5439), *Bacillus cereus* (RDK 106, WDCM 00001), *Staphylococcus aureus* (RDK 056, WDCM 000034), methicillin-resistant *S. aureus* (MRSA 1.4, EQAS 2009), and *Listeria monocytogenes* (RDK 059). A bacterial suspension equivalent in density to the 0.5 McFarland turbidity standard was prepared and spread on Mueller–Hinton agar plates (Sigma-Aldrich, Germany). After 5 min, the round biofoil disks (approximately 5.5 mm in diameter) were placed on the inoculated agar and incubated at 37 °C for 24 h. The tetracycline disk (30 µg, BBL Becton Dickinson, Franklin Lakes, NJ, USA) was used as a positive control, and the disk without JKRB was used as a negative control. After incubation, the inhibition zones (the growth-free zones around the disk) were measured in mm using a vernier caliper. Each bacterial strain was tested with 12 replicates, and the data are given as the mean values with the standard deviation.

### 2.6. Physicochemical Properties of the Biofoils

ATR-FTIR analyses of JKRB, chitosan powder, and chitosan (blank) and JKRB biofoils were carried out to observe the interaction between the compounds. The spectra were recorded on Spectrum Two (Perkin Elmer, Rodgau, Germany) from wavenumber 4000 cm^−1^ to 400 cm^−1^ with a step of 4 cm^−1^, accumulating 32 scans.

Thicknesses were measured at five different positions of the chitosan and JKRB biofoils using a calibrated ABS Digital Thickness Gauge (Mitutoyo, Aurora, IL, USA), and the results were averaged.

The biofoils were mechanically characterized according to guidelines of the ASTM D882 standard method [40]. The tensile strength (TS) and elongation at break (EB) were determined simultaneously by testing rectangular biofoil samples (length × width = 8 cm × 2 cm; gage length segment 6 cm) on the XLW Auto Tensile Tester (Labthink^®^ Instruments, Jinan, China) equipped with a 100 N load cell, at a crosshead speed of 25 mm min^−1^. The calculations for both parameters were performed using a Lystem^TM^ Lab Data Sharing System.

Moisture content (MC), swelling degree (SD), and total soluble matter (TSM) were determined gravimetrically. The rectangular biofoil samples (~1 cm^2^) were weighed on an analytical balance (Kern & Sohn, Balingen, Germany) with a precision of 0.0001 g in order to determine the initial mass (M_1_). The samples dried in the oven at 105 °C for 24 h to obtain the initial dry mass (M_2_) were separately immersed in water (30 mL) and left at room temperature for 24 h. The excess water that was not absorbed by the biofoil samples was superficially removed using paper wipes, and the samples were weighed again to determine the wet mass of the biofoil (M_3_). The samples were dried again at 105 °C for 24 h to obtain the final dry mass (M_4_). The experiments were performed in triplicate.

MC (Equation (2)) was defined as the percentage of mass loss due to water evaporation after drying at 105 °C for 24 h. SD (Equation (3)) was defined as the percentage of mass gain due to the water absorption after dry sample immersion in water for 24 h. TSM (Equation (4)) was defined as the percentage of the biofoil dry matter that was solubilized after 24 h of immersion in water:

MC = ((M_1_ − M_2_)/M_1_) × 100% (2)

SD = ((M_3_ − M_2_)/M_2_) × 100% (3)

TSM = ((M_2_ − M_4_)/M_2_) × 100% (4)

Total phenolic content (TPC) was estimated using Folin–Ciocalteu’s phenol reagent. Small, pre-weighted, rectangular biofoil samples were placed in glass vials, to which water was added to reach the final biofoil concentration of 5.00 mg mL^−1^, followed by the successive addition of Folin–Ciocalteu’s phenol reagent and 10% (*w/v*) aqueous solution of Na_2_CO_3_ (10% and 20% were added based on the volume of water, respectively). After the sample incubation for 2 h (dark conditions; room temperature), the absorbance of the solutions was measured at 765 nm using the Synergy™ 2 Multi-Detection Microplate Reader (BioTek, Winooski, VT, USA). The results were expressed as the mass of gallic acid equivalent (GAE) per mass of the biofoil.

### 2.7. Investigation of Biofoils’ Biodegradability

The degradation of the chitosan (blank) and JKRB biofoils was assessed in industrial compost, supplied by a local waste management facility. Compost (about 200 g) was weighed into a crystallizing dish with a diameter of 13.5 cm. Eight pieces of chitosan biofoil measuring 2 cm × 2 cm with known masses were placed on the previously marked areas in the compost so that they were completely covered. The crystallizing dish was covered with Parafilm M^®^, which was punctured with a needle approximately ten times to ensure air circulation. The samples were collected over a period of 11 days. Biofoil residues were carefully removed with tweezers, washed with distilled water so that there was no soil debris on the surface, and dried to constant masses in a ventilation oven (Kambič, Semič, Slovenia) at 105 °C, with 30% ventilation. Biodegradation was calculated as described in Ref. [25]. The moisture content of the soil was measured with the HE53 Moisture Analyzer (Mettler Toledo, Columbus, OH, USA).

## 3. Results and Discussion

### 3.1. Antioxidant Activity of the Biofoils

#### 3.1.1. DPPH Scavenging Activity of Food Simulants after Contact with the Biofoils

The extraction of lyophilized and pulverized Japanese knotweed rhizome bark was performed with eight different solvents and solvent mixtures (distilled water, ethanol, 70% ethanol_(aq)_, methanol, 80% methanol_(aq)_, acetone, 70% acetone_(aq)_, and 90% ethyl acetate_(aq)_) [17]. The extraction yield was the highest with 70% ethanol_(aq)_ [17] and increased from 44.0% with a single extraction [17] to 51.5% with a double extraction (the present study). The JKRB 70% ethanol_(aq)_ extract showed strong antioxidant activity (IC_50_ = 3.50 µg mL^−1^; DPPH assay), which was in the range of the antioxidant activity of the known antioxidant, ascorbic acid (vitamin C) [17]. Moreover, the antioxidant activity of JKRB was stable for at least 14 days, which was not the case with vitamin C (it decreased over time) [17]. Additionally, 70% ethanol_(aq)_ is considered a solvent of low toxicity, suitable for use in pharmaceutical and food industries, and can be obtained as a food-grade solvent.

Considering these facts, the dry 70% ethanol_(aq)_ extract was incorporated into a chitosan-based biofoil with the final aim to be used as packaging for food/beverages, food supplements, medicines, or cosmetics and to protect the container material and/or the packed content from oxidation. In the case that a sufficient amount of antioxidant extract is incorporated into the biofoils, the food in contact with the potentially active biofoil will be enriched with antioxidants, thus forming functional, antioxidant food.

To study the antioxidant activity of the formulated biofoil, the chitosan (blank) and JKRB biofoils were exposed to liquid food simulants (10% ethanol_(aq)_ (*v/v*), 3% acetic acid_(aq)_ (*w/v*), 20% ethanol_(aq)_ (*v/v*), 50% ethanol_(aq)_ (*v/v*), and 95% ethanol_(aq)_ (*v/v*)), selected according to the European Commission’s document No 10/2011 [36]. Vegetable oil is a simulant of choice for lipophilic foods and foods with free fats at the surface [36]. However, it possesses antioxidant activity itself and masked the effect of the biofoil (DPPH assay); therefore, 95% ethanol_(aq)_ (*v/v*) was used as its substitute.

**Figure 4:**
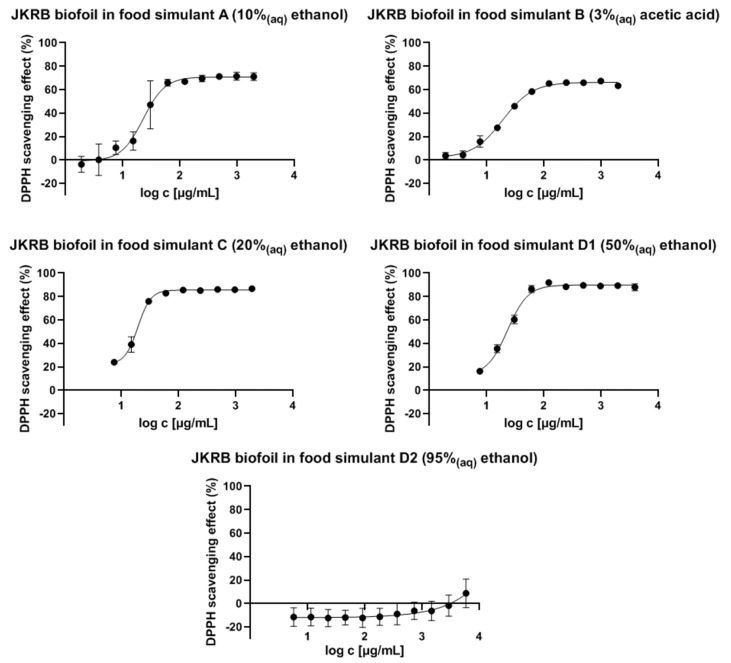
Logarithmic curves of the DPPH radical scavenging effect (%) relative to the “concentration” of JKRB biofoil in the liquid food simulants (graphs plotted using GraphPad Prism 9; Sigmoidal, 4PL fit).

A spectrophotometric DPPH assay (example shown in Figure 3) demonstrated the antioxidant activity of the food simulants in contact with the JKRB biofoil, while the food simulants in contact with the chitosan (blank) biofoil did not show antioxidant activity. The antioxidant activity was confirmed with all tested liquid food simulants (A, B, C, and D1), except with 95% ethanol_(aq)_ (simulant D2) after exposure to JKRB biofoil. The IC_50_ values (mg mL^−1^; theoretical “concentration” of active biofoil relative to the volume of liquid food simulant) were determined using GraphPad Prism 9 software, San Diego, CA, USA [38] (Figure 4 and Table 1).

The antioxidant activity (IC_50_ values; Table 1) of the liquid food simulants upon contact with the JKRB biofoil was a consequence of the expected leaching of the polar compounds of JKRB into food simulants with a high percentage of water (≥50%).

However, a compromise between the antioxidant activities and physicochemical properties of the biofoil should be made to select the most appropriate type of food for packing.

#### 3.1.2. Migration of the Antioxidant Marker, (−)-Epicatechin, from the Biofoils into Food Simulants

##### LC-MS Analysis

**Figure 5:**
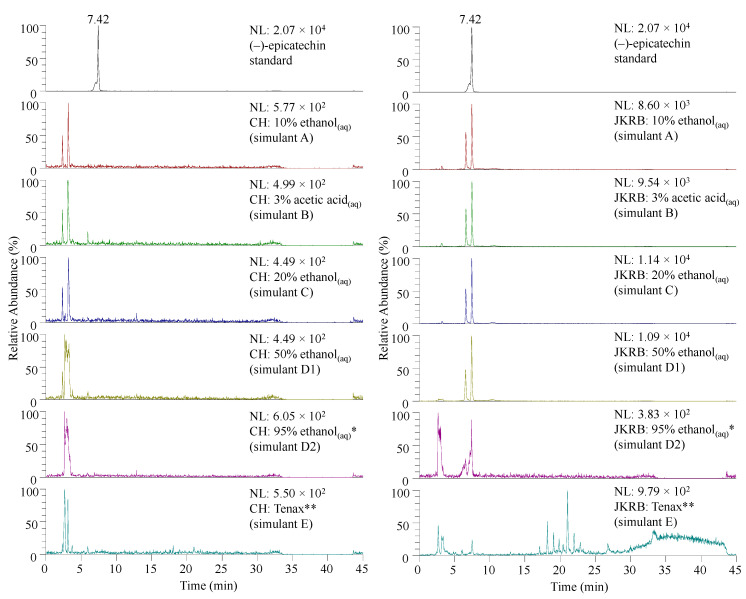
SIM chromatograms of (−)-epicatechin (*t_r_* at 7.42 min) acquired for stock solutions of liquid food simulants (about 77 mg of biofoil per 20 mL of food simulant after 1 h of contact) analyzed directly, except simulant D (3-fold concentrated) * and extract of simulant E (10-fold concentrated) **. SIM chromatogram of the standard (−)-epicatechin is shown at the top of each column for comparison. Abbreviations: CH—chitosan biofoil; JKRB—JKRB biofoil.

**Figure 6:**
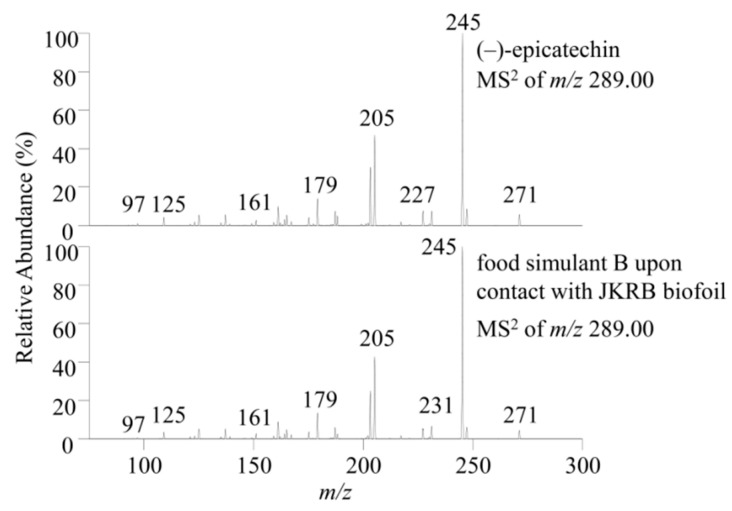
Comparison of MS^2^ spectra of (−)-epicatechin standard and the target compound in the sample (a leachable of JKRB biofoil into food simulant B (3% acetic acid_(aq)_) is given as an example). (−)-Epicatechin was identified in all food simulants based on *t_r_*, MS, and MS^2^ spectra.

In a recent study, (−)-epicatechin was determined as a compound that importantly contributes to the antioxidant activity of JKRB, according to the online DPPH-guided HPLC-SEC-UV/Vis fractionation. This was followed by compound identification using RP-HPLC-UV and high-performance thin-layer chromatography and final identity confirmation with a reference standard [17]. Therefore, (−)-epicatechin was selected as a marker of the antioxidant activity of JKRB and its migration from the chitosan (blank) and JKRB biofoils into food simulants (all liquid food simulants from Table 1 and Tenax, a porous polymer which simulates dry foods [36]) was tested using the LC-MS method [17] using a C18 column. (−)-Epicatechin was identified in the food simulants that were in contact for 1 h with JKRB biofoil using the SIM mode (*m/z* 289) and MS^2^ (Figure 5 and Figure 6, respectively, and Table 1), and as expected, was not detected in food simulants upon 1 h of contact with chitosan (blank) biofoil (Figure 5).

Although (−)-epicatechin migrated into the oily food simulant (95% ethanol_(aq)_), as observed from the LC-MS results (Figure 5), the “concentration” of the JKRB biofoil needed to scavenge 50% of DPPH free radicals was much higher than the experimental “concentrations”, as predicted using GraphPad Prism (Figure 4).

LC-MS analyses confirmed that (−)-epicatechin from the JKRB biofoil also migrated into the dry food simulant (Tenax). As the observed mass peak (SIM mode) of (−)-epicatechin was of much lower intensity (<LOQ) than in the case of other simulants, the extract was 10-fold concentrated for quantitative analysis. The direct comparison of mass peak intensities was not possible since the sample preparation for the Tenax simulant differed from the one for the liquid simulants.

The LC-MS method for the quantification of (−)-epicatechin in food simulants was validated for the following parameters: range, linearity, LOD, LOQ, repeatability, accuracy, and precision. The concentration range was from 0.25 to 25.00 µg mL^–1^, R^2^ was 0.9998, LOD and LOQ were 0.05 µg mL^–1^ and 0.10 µg mL^–1^, respectively, and the RSD% of the (−)-epicatechin peak area at 2.50 µg mL^–1^ (*n* = 6) was 3.9%. The accuracy of the method measured in triplicate at levels 0.50, 2.50, and 5.00 µg mL^–1^ was 124%, 112%, and 97%, respectively, while precision for the same levels in six replicates was 5.2%, 2.3%, and 3.7%, respectively. The acceptance criteria for the accuracy of this method were set from 80 to 120% and only for the lowest concentration from 70 to 125% [41]. As an MS detector was used for the quantification (SIM mode) and confirmation of the identity of the target compound (MS^2^), the method was considered to be specific. A correlation was found between the concentrations of (−)-epicatechin in the food simulants upon contact with JKRB biofoil (Table 2) and the antioxidant activities of the studied food simulants (IC_50_ values; Table 1). Namely, food simulants with low IC_50_ values of antioxidant activities showed high concentrations of (−)-epicatechin, while the oily food simulant (95% ethanol_(aq)_) for which a high IC_50_ value was predicted using GraphPad showed a significantly lower concentration of (−)-epicatechin. This supports the claim that (−)-epicatechin is a marker of the antioxidant activity of JKRB.

Concerning the antioxidant activity, the mechanisms of action of proanthocyanidins and their monomers, flavan-3-ols, to which epicatechin belongs, include free radical scavenging, transition metals chelation [42,43], and enzyme inhibition [43]. On a molecular level, they effectively suppress oxidative stress through the mediation of several molecular targets (e.g., DNA damage repair and lipid peroxidation prevention) and the modulation of some specific signaling pathways [44]. (–)-Epicatechin is supposed to act as an antioxidant as a free radical scavenger [45], described as a highly effective quencher of hydroxyl and superoxide radicals [42], and indirectly as a modulator of glutathione peroxidase and superoxide dismutase [45].

##### Calculations of the Migration of the Antioxidant Marker, (−)-Epicatechin

Using the LC-MS method, the concentration of (−)-epicatechin in JKRB was determined to be 13.91 µg mL^–1^, which represented 1% *w/w* of (−)-epicatechin in the extract (1.35 mg mL^−1^ JKRB prepared in methanol). The calculations showed that after 1 h of contact, cca. 87% of (−)-epicatechin incorporated into the biofoil leached from the JKRB biofoil into the food simulants A, B, C, and D1 (average concentration of 6.61 µg mL^−1^ (Table 2)), meaning an average of 132 µg of (−)-epicatechin out of 151.68 µg incorporated (−)-epicatechin (1% *w/w* of 15.17 mg of JKRB corresponding to the stock solutions, see Calculations of the Migration of the Antioxidant Marker, (−)-Epicatechin in Section 2.4.2) was leached into the 20 mL stock solutions. A time-dependent release of the antioxidant might have been provided by modification of the formulation (e.g., the encapsulation of the extract into an additional polymer [46] before its incorporation into a chitosan matrix). However, JKRB, as well as its marker of antioxidant activity, (−)-epicatechin, showed stable antioxidant activity for at least 14 days, [17] which might provide an alternative to the gradual release of the antioxidant into the food simulants.

Moreover, the calculations show that 1 cm^2^ of the JKRB biofoil provided antioxidant activity to cca. 0.5 L of liquid food (4.2 mL (see Calculations of the Migration of the Antioxidant Marker, (−)-Epicatechin in Section 2.4.2) multiplied by a dilution factor of cca. 128—a dilution of stock solution which possesses the closest biofoil “concentration” to (or slightly above) the IC_50_ value showing antioxidant activity upon 1 h of biofoil contact with food simulants A, B, C, and D1; Section 3.1.1). Taking the above numbers into account, 1 cm^2^ of JKRB biofoil would leach cca. 30 µg of (−)-epicatechin into approximately 0.5 L of liquid food.

For comparison, the no observed adverse effect level (NOAEL) of green tea catechins ((−)-epicatechin and others) was estimated to be 764 mg/kg/day and 820 mg/kg/day for male and female rats, respectively [47], while for resveratrol (also a known phytochemical of JKRB), it was calculated to be 200 mg/kg/day in rats and 600 mg/kg/day in dogs [48]. From here, acceptable daily intake (ADI) can easily be calculated by dividing the NOAEL value by an uncertainty (safety) factor of 100. 

The overall migration limit (OML) of 10 mg per dm^2^ in the case of the deliberate release of the active agent from the biofoil is allowed to be exceeded when the active function is not a specific feature of the passive material. In this case, the released amount of active agent should not be included in the OML evaluation [21]. Moreover, chitosan biofoil is considered edible [49], while at the same time, dietary supplements with extracts of Japanese knotweed rhizome are already sold in pharmacies and specialty stores. Nevertheless, it is necessary to be aware of the lack of toxicological data on this plant material. For example, its safe use for pregnant and lactating women has not been established yet [50].

Taking into account the confirmed migration of the JKRB antioxidants from the biofoil into food simulants, JKRB extract can further be incorporated into other “plastic materials”, gelatin capsules, and other biodegradable materials to protect packed goods or packaging material itself, thus potentially increasing their stability and shelf life. The biopolymers with incorporated JKRB may potentially be useful for:

(a) active packaging (e.g., packaging for food, cosmetics, pharmaceuticals, and food supplements): JKRB would protect both the content and the packaging from oxidation. Antioxidants would enrich the goods by migrating from the packaging into the contact content, thereby, in the case of food, indirectly providing functional antioxidant food/beverages;
(b) the protection of various rapidly oxidizing materials from oxidation (e.g., industrial fluids): the migration of JKRB antioxidants from the polymeric material into the fluid may be regulated by the amount of extract and various additives incorporated into the biopolymer;
(c) products that come into contact with skin (e.g., face masks, patches, etc.): the migration of JKRB antioxidants into skin simulants should additionally be tested;
(d) food (e.g., edible antioxidant chitosan-JKRB gummy bears).

Additional studies should be conducted to confirm the safety of such products.

### 3.2. Antimicrobial Activity of the Biofoils

Foodborne bacteria are considered a major concern for food safety and may lead to food spoilage or food poisoning. They can seriously affect the quality of food during all stages of production. Therefore, the antibacterial property is very important for food packaging biofoils [51]. In the present study, the antimicrobial activity of chitosan (blank) and JKRB biofoils was tested against nine foodborne pathogens. The in vitro evaluation of the antimicrobial activity of the JKRB biofoil revealed that it inhibits Gram-positive bacteria more efficiently than Gram-negative bacteria (Table 3). The inhibitory zones of the JKRB biofoil were clearly observed for all tested Gram-positive bacteria, including *B. cereus*, *S. aureus*, and MRSA, except for *L. monocytogenes*. The latter grew to the edge of the biofoil disk; however, a bacteriostatic effect was observed against it (smaller colonies of *L. monocytogenes*). On the contrary, no inhibition zones were found in the case of Gram-negative bacteria (*E. coli*, ESBL, *S*. Enteritidis, *S*. Typhimurium, and *Y. enterocolitica*). The negative control (chitosan biofoil) did not show any inhibition zone, and the positive control (tetracycline) showed an inhibition zone in the range of 21–33 mm, dependent on the bacteria.

Although chitosan is known for its antimicrobial activity, these properties are only exhibited if the matrix pH is lower than chitosan’s pKa (ranging from 6.2 to 7.0), making chitosan a polycationic species and thus enabling electrostatic interactions with negatively charged cell envelope structures and cytoplasmic membrane. Gram-negative bacteria have been shown to be more susceptible to chitosan than Gram-positive bacteria [52]. On the contrary, Japanese knotweed rhizome and herb extracts, although active against both Gram-positive and Gram-negative bacteria, have shown higher susceptibility against Gram-positive bacteria [2,18,53]. The proposed mechanism of action of some Japanese knotweed extracts includes activity against the cell membrane or extracellular proteins, resulting in bacterial cell apoptosis [53]. The literature findings support the results obtained for JKRB biofoil, which showed activity against Gram-positive bacteria. The incorporation of a higher % of JKRB into a chitosan-based biofoil in the future may enhance the biofoil’s antibacterial activity and potentially lead to activity even against Gram-negative bacteria.

### 3.3. Physicochemical Properties of the Biofoils

**Figure 7:**
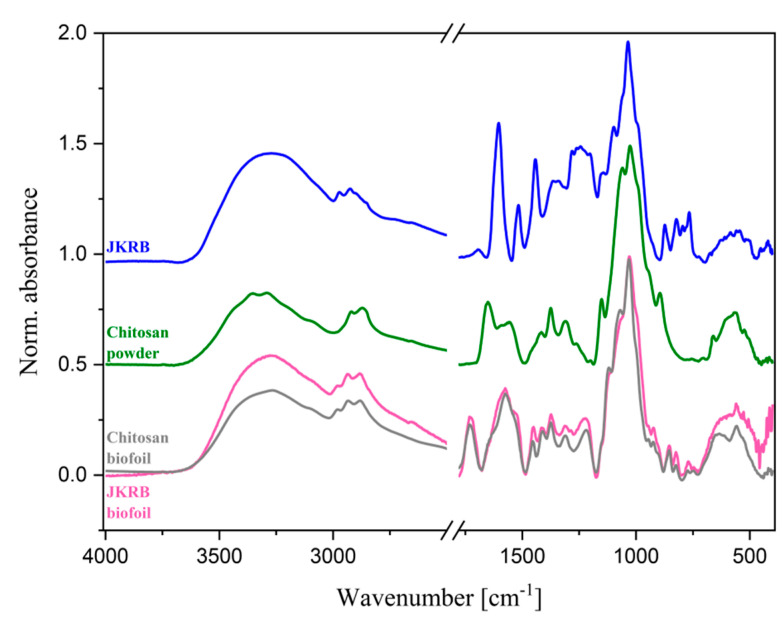
ATR-FTIR spectra of JKRB, chitosan powder, and chitosan (blank) and JKRB biofoils.

To observe the interactions between the compounds in JKRB biofoil, ATR-FTIR analysis was carried out. Firstly, JKRB and chitosan powder were studied separately. The spectrum of the JKBR extract (Figure 7) revealed a broad peak positioned between 3690 cm^−1^ and 2996 cm^−1^ corresponding to the O-H stretching of free alcohols. A smaller peak in the region between 2990 cm^−1^ and 2800 cm^−1^ indicated the presence of C-H stretching. Furthermore, sharp signals were observed at 1603 cm^−1^, 1510 cm^−1^, 1447 cm^−1^, and 1032 cm^−1^ that were correlated to C=C stretching in cyclic alkene, N-O stretching, O-H bending, and C-O stretching vibrations, respectively. A series of peaks between 870 cm^−1^ and 766 cm^−1^ were associated with C=C bending in alkene. The spectrum of chitosan (Figure 7) revealed its characteristic peaks that were in agreement with the published literature [26]: a broad peak in the region between 3600 cm^−1^ and 3200 cm^−1^ corresponding to O-H and N-H stretching, a peak positioned at 2925 cm^−1^ (C-H stretching), absorption peaks correlated with C=O stretching in amide I (positioned at 1653 cm^−1^), N-H bending in amide II (centered at 1556 cm^−1^), C-N stretching in amide III (1372 cm^−1^), and C-O stretching vibrations (1023 cm^−1^). Comparing the pure spectra of chitosan powder and chitosan biofoil (Figure 7), structural changes upon film formation could be observed, mainly visible in the region between 1770 cm^−1^ and 1173 cm^−1^ (corresponding to amide I, amide II, and amide III), due to chitosan–glycerol interactions and the protonation of the amino group in acidic conditions after the addition of lactic acid [26,54]. The incorporation of JKRB into the chitosan biofoil caused a slight change in several regions in an otherwise similar spectrum, mostly by intensifying the signals in regions corresponding to O-H, N-H, and C-H stretching (between 3700 cm^−1^ and 2670 cm^−1^), suggesting the possible formation of hydrogen bonds between chitosan’s N-O and O-H groups and the functional groups of the JKRB extract’s compounds, such as (–)-epicatechin’s OH groups [55]. Furthermore, increased absorbance was found at 1453 cm^−1^, 1308 cm^−1^, and 1216 cm^−1^, as well as in the region between 882 cm^−1^ and 724 cm^−1^.

The determined physicochemical properties of the chitosan (blank) and JKRB biofoils revealed that the addition of JKRB into chitosan biofoil elevated the biofoil’s physical integrity, water resistance, and chemical activity approximately 50% at most towards a positive outcome, variable within different parameters (Table 4).

The chitosan and JKRB biofoils showed statistically indistinguishable thickness, indicating homogeneous extract dispersion into the matrix. This is not the case with all plant extracts; for example, in a previous study, a chestnut extract, which was incorporated into a biofoil in the same ratio as JKRB, increased the biofoil’s thickness compared to the control with an average of 120 ± 2 μm [26]. This could be related to the chemical composition of the extract, as chestnut extract may have a higher fiber content (prone to clumping from certain concentrations) than JKRB and, certainly, to the different extraction procedures, which implies variability in the particle sizes of the dry extract powder. Other studies [35,56] affirm uniform preparation on a lab scale, thus supporting previous statements. It was impossible to pinpoint exactly how the authors of the Ref. [57] achieved the total homogeneity of the biofoils with added orange peel extract, which was 17 ± 2.6 μm thicker compared to the control, and the extract was described to be rich in pectin, hemicellulose, limonene, etc. This supports the importance of understanding the influence of the chemical composition and the particle size of the dry extract powder on biofoil thickness. Apart from macromolecular disparities, the result for JKRB biofoil of 120 μm corresponds to almost half of the medical scaffold thickness (starting from 300 μm for animals) [58,59] and could be considered as a good food packaging material thickness in terms of integrity [60,61].

In comparison to neat polylactic acid foils, a biofoil is considered to have good performance when its tensile strength varies between 24.8 and 70.2 MPa [62], depending on the application (more for food packages and less for medical scaffolds). Relative to the chitosan (blank) biofoil, the JKRB biofoil with incorporated extract in a concentration of 0.01 mg mL^−1^ (1%) did not display outstanding performance with regard to tensile strength, only increasing it by up to 1.81 MPa, which was much lower than that achieved using chestnut extract (0.01 mg mL^−1^, 15.6 MPa) [35] or curcumin extract (0.08 mg mL^−1^, 18.8 MPa) [63].

On the other hand, JKRB incorporation into the biofoil decreased the elongation at break down to 27.7%, making the biofoil stretchy enough, which is important for food packaging intended for heavier products (e.g., meat or cheese). Previously, it was shown that a biofoil (1% tannic acid: chitosan, 144 cm^2^) suitable to pack 22 g of cheese should have an elongation at break of approximately 28.5% to withhold without breaking apart [35], and JKRB biofoil corresponds to this result. A recent reference has shown this value to be 57.0% to prepare a high-performance scaffold [64], while this parameter could ideally be lower. The range for the elongation at break for polylactic-acid-based materials has been shown to be between 1.0 and 7.9% [62], which is unlikely to be achieved using cyclic polymers such as chitosan.

As expected, the incorporation of JKRB into chitosan biofoil decreased the initial moisture content (MC) value compared to the control. This can be explained by the interactions in the first stages of the biofoil preparation, where chitosan polymers cross-link with polyphenols and fibers of the extract [65], which was confirmed with ATR-FTIR, leaving less interaction possibility for water and facilitating its evaporation during the drying phase.

The swelling degree of the JKRB biofoil remained high, although it was possible to increase the JKBR concentration in the biofoil by up to 8% without affecting other parameters, which may have helped to decrease the swelling rate, as shown in Ref. [63]. The total soluble matter concentration effectively describes how the blank biofoil in a water environment swells up to its capacity and gradually solubilizes into an uniform mass, leaving behind a gel-like texture [66], while JKRB biofoil retains swelling, and seemingly at some point starts to solubilize to a higher extent than the blank. Some authors [66,67] considered this to be due to the hydrophilicity of polyphenolic content and chitosan itself when interacting with water.

Supporting the results of the DPPH analyses (Section 3.1.), the TPC analyses confirmed biofoils to be ~2% more antioxidative than a previously studied biofoil with the addition of 1% chestnut extract [28].

### 3.4. Investigation of Biofoils’ Biodegradability

**Figure 8:**
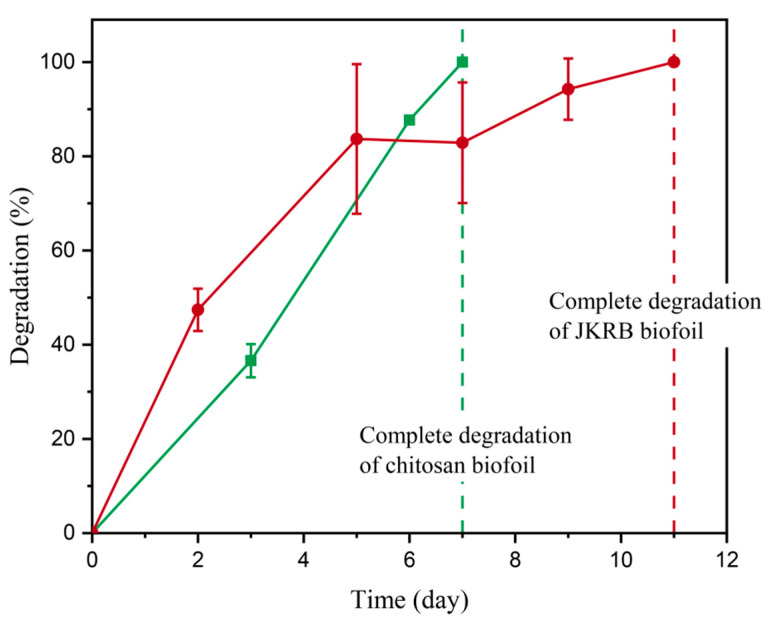
Degradation of chitosan (blank) biofoil (green curve) and JKRB biofoil (red curve) in industrial compost.

Chitosan (blank) and JKRB biofoils were exposed to animate and inanimate activity in industrial compost with a moisture content of 49.0 ± 4.0%. The JKRB biofoil degraded over a period of 11 days, while the chitosan biofoil fully deteriorated in 7 days (Figure 8), which was in accordance with the previous studies [29]. The antioxidant properties and the repellent effect on microorganisms obtained via the incorporation of JKRB were most possibly responsible for the longer degradation rate of JKRB biofoil. However, in the first five days, the degradation of the JKRB biofoil was faster than the chitosan biofoil, which could be attributed to the higher proportion of water-soluble matter which is responsible for fast mass loss in the initial stages of degradation. Further, the antioxidant extract disrupts the hydrogen bonding between chitosan chains by establishing new bonds with JKRB, which results in lower density, thus making the surface more susceptible to the attack of microorganisms. During the process of degradation, the material is first mechanically disintegrated into fragments which are then mineralized by microorganisms or further fragmented under UV and thermal influence without final conversion to inorganic carbon. The identification and quantification of the latter are out of the scope of the applied method.

## 4. Conclusions

To date, this is the first study that exploited the invasive alien plant species, Japanese knotweed, as a natural antioxidant and preservative for polymers. It was shown that two biowastes, 1. chitosan, derived from crustacean shell waste and 2. Japanese knotweed rhizome bark, a leftover after the mechanical excavation of the plant material, can be transformed into active biofoil.

The antioxidant activity of the liquid food simulants (A, B, C, D1, and D2) upon 1 h of contact with the JKRB biofoil was demonstrated for the food simulants A, B, C, and D1 using a DPPH spectrophotometric assay (measured IC_50_).

The migration of the antioxidant marker, (−)-epicatechin, from the JKRB biofoil into all tested food simulants (A, B, C, D1, D2, and E) was confirmed and quantified using a validated LC-MS method, although its quantity was higher in the liquid food simulants A, B, C, and D1.

About 87% of the antioxidant marker, (−)-epicatechin, was released from the JKRB biofoil into the liquid food simulants A, B, C, and D1, in 1 h. However, a previous study showed the stable antioxidant activity of (−)-epicatechin over time, which might provide an alternative to a gradual release of antioxidants into food.

The calculations also showed that 1 cm^2^ of JKRB biofoil is able to provide antioxidant activity to approximately 0.5 L of liquid food, implying the potent antioxidant activity of JKRB biofoil. 

JKRB biofoil (JKRB 1% *w/v*) showed antimicrobial activity against Gram-positive bacteria (*B. cereus*, *S. aureus*, including MRSA, and *L. monocytogenes*).

The tensile strength and TPC improved upon the addition of JKBR into the biofoil, while the elongation at break and swelling degree decreased. The physicochemical properties of the JKRB biofoil make it more suitable for packing heavier products (e.g., meat and cheese).

ATR-FTIR analysis revealed the formation of hydrogen bonds between chitosan biofoil’s and JKRB extract’s compounds. Lastly, the JKRB biofoil completely degraded in industrial compost within 11 days.

The future upscaled production of JKRB biofoils may contribute to solving several ecological and economic problems in the following ways: (i) re-using biowaste, such as shrimp leftovers from restaurants and Japanese knotweed plant waste left after its mechanical eradication; (ii) aiding the removal of the invasive alien plant species, Japanese knotweed, from the environment and consequently protecting autochthonous biodiversity; (iii) replacing conventional plastics with biopolymer plastics, which decompose rapidly in compost, helping to reduce greenhouse gasses; (iv) extending the shelf life of packed food and other products (due to antimicrobial and antioxidant activity), which leads to reducing food waste.

## 5. Patents

Slovenian patent application: P-202100115, Slovenian Intellectual Property Office, filed 03.06.2021.

## Figures and Tables

**Table 1:** Antioxidant activity of the food simulants, selected according to the European Commission’s document No 10/2011 [36], upon 1 h of contact with the formulated JKRB biofoil, and migration of the selected marker of the antioxidant activity, (–)-epicatechin, from the JKRB biofoil into food simulants. The food simulants in contact with the chitosan (blank) biofoil did not show antioxidant activity, except for food simulant E (not measured).

Food Simulant	Assignment of Food Simulant to Food	Mass of Biofoil in Volume of Simulant (mg mL^−1^)	IC_50_ (µg mL^–1^)	Hillslope	LC-MS; (–)-Epicatechin SIM [*m/z* 289], MS^2^
**A**: 10% ethanol_(aq)_ (*v/v*)	Hydrophilic foods	77/20	22.72 (17.36–28.34)	2.27 (1.37–4.08)	+
**B**: 3% acetic acid_(aq)_ (*w/v*)	Hydrophilic foods, pH below 4.5	77/20	18.99 (16.85–21.24)	1.68 (1.44–1.97)	+
**C**: 20% ethanol_(aq)_ (*v/v*)	Hydrophilic foods with a relevant amount of organic ingredients, foods with an alcohol content of up to 20%	77/20	19.40 (18.05–20.91)	3.85 (3.12–4.72)	+
**D1**: 50% ethanol_(aq)_ (*v/v*)	Lipophilic foods, foods with alcohol content of above 20%, oil in water emulsions (e.g., milk).	77/20	23.16 (20.18–25.99)	2.44 (2.01–2.97)	+
**D2**: 95% ethanol_(aq)_ (*v/v*)	Lipophilic foods, foods with free fats at the surface	231/20	/		+
**E**: Tenax	Dry foods	*	not measured		+

* 160 mg of Tenax powder / 4 cm^2^ of biofoil --> extraction with 2 × 3 mL methanol --> concentration to 1 mL of methanol.

**Table 2:** (−)-Epicatechin (µg mL^–1^) quantified in food simulants upon contact with JKRB biofoil. (−)-Epicatechin was not detected in food simulants upon contact with the chitosan (blank) biofoil. Stock solutions of all liquid food simulants were directly analyzed, while 95% ethanol_(aq)_ (*v/v*) was 3-fold concentrated *, and Tenax extract was 10-fold concentrated **.

Food Simulant	(−)-Epicatechin (µg mL^–1^)
A: 10% ethanol_(aq)_ (*v/v*)	5.23
B: 3% acetic acid_(aq)_ (*w/v*)	6.66
C: 20% ethanol_(aq)_ (*v/v*)	7.00
D1: 50% ethanol_(aq)_ (*v/v*)	7.54
D2: 95% ethanol_(aq)_ (*v/v*) *	0.46
E: Tenax **	0.58

**Table 3:** Antimicrobial activity of the JKRB biofoil against Gram-positive and Gram-negative bacteria. Values are expressed as mean ± SD (*n* = 12).

Microorganism	Diameter of Inhibition Zone (mm)
**Gram-positive bacteria**	
*B. cereus*	2.92 ± 1.00
*S. aureus*	1.93 ± 1.21
MRSA	1.49 ± 0.85
L. monocytogenes *	3.18 ± 1.14
**Gram-negative bacteria**	
*E. coli*	0.00 ± 0.00
ESBL	0.00 ± 0.00
*S*. Enteritidis	0.00 ± 0.00
*S*. Typhimurium	0.00 ± 0.00
*Y. enterocolitica*	0.00 ± 0.00

* bacteriostatic effect.

**Table 4:** Modified physicochemical properties of the JKRB biofoil in comparison to the chitosan (blank) biofoil.

	JKRB Biofoil	Chitosan (Blank) Biofoil
Thickness (µm)	120.00 ± 1.00	110.00 ± 1.00
Tensile strength (MPa)	1.81 ± 0.36	0.75 ± 0.33
Elongation (%)	27.70 ± 6.52	62.80 ± 9.16
Moisture content (%)	16.90 ± 0.27	25.60 ± 1.29
Swelling degree (%)	20.30 ± 0.39	32.60 ± 2.30
Soluble mass fraction (%)	38.40 ± 5.77	31.30 ± 0.75
Content of all polyphenols (mg_GAE_ g_foil_^−1^)	20.50 ± 0.44	2.36 ± 0.61

## Data Availability

All data generated for this study are included in the article.
